# Head and neck small-cell carcinoma: A multicenter study of 39 cases from 10 institutions

**DOI:** 10.3389/fsurg.2022.1049116

**Published:** 2022-11-14

**Authors:** Hiroshi Matsuyama, Yushi Ueki, Isaku Okamoto, Toshitaka Nagao, Kohei Honda, Keisuke Yamazaki, Ryuichi Okabe, Takafumi Togashi, Ryusuke Shodo, Hisayuki Ota, Takeshi Takahashi, Jo Omata, Yusuke Yokoyama, Kohei Saijo, Ryoko Tanaka, Kiyoaki Tsukahara, Tadashi Kitahara, Hirokazu Uemura, Seiichi Yoshimoto, Fumihiko Matsumoto, Kenji Okami, Akihiro Sakai, Kenichi Takano, Atsushi Kondo, Hidenori Inohara, Hirotaka Eguchi, Nobuhiko Oridate, Teruhiko Tanabe, Munenaga Nakamizo, Kazuhiko Yokoshima, Koki Miura, Yosuke Kitani, Arata Horii

**Affiliations:** ^1^ Department of Otolaryngology, Niigata City General Hospital, Niigata, Japan; ^2^ Department of Otolaryngology, Head and Neck Surgery, Niigata University Graduate School of Medical and Dental Sciences, Niigata, Japan; ^3^ Department of Otolaryngology, Head and Neck Surgery, Tokyo Medical University, Nishishinjuku, Japan; ^4^ Department of Anatomic Pathology, Tokyo Medical University, Nishishinjuku, Japan; ^5^ Department of Otolaryngology-Head and Neck Surgery, Nara Medical University, Kashihara, Japan; ^6^ Department of Head and Neck Surgery, National Cancer Center Hospital, Tsukiji, Japan; ^7^ Department of Otorhinolaryngology, Juntendo University Faculty of Medicine, Isehara, Japan; ^8^ Department of Otolaryngology-Head and Neck Surgery, Tokai University School of Medicine, Sapporo, Japan; ^9^ Department of Otolaryngology, Sapporo Medical University School of Medicine, Suita, Japan; ^10^ Department of Otorhinolaryngology-Head and Neck Surgery, Osaka University Graduate School of Medicine, Yokohama, Japan; ^11^ Department of Otorhinolaryngology, Head and Neck Surgery, School of Medicine, Yokohama City University, Sendagi, Japan; ^12^ Department of Otorhinolaryngology, Nippon Medical School Hospital, Mita, Japan; ^13^ Department of Otorhinolaryngology, Tokyo Women’s Medical University, Hongo, Japan; ^14^ Department of Head and Neck Surgery, Tochigi Cancer Center, Kawada, Japan; ^15^ Department of Head and Neck Oncology and Surgery, International University of Health and Welfare Mita Hospital, Utsunomiya, Japan

**Keywords:** small-cell carcinoma, neuroendocrine carcinoma, head and neck carcinoma, concurrent chemoradiotherapy, diagnostic and therapeutic algorithms

## Abstract

**Objective:**

Basal information of head and neck small-cell carcinoma (HNSmCC) including epidemiology, primary site, treatment, and prognosis remains sparse due to its rarity. We report here a multicenter retrospective study on the diagnosis, treatment, and outcomes of patients with HNSmCC.

**Materials and methods:**

This study involved 47 patients with HNSmCC from 10 participating institutions. Eight patients were excluded for whom no pathological specimens were available (*n* = 2) and for discrepant central pathological judgements (*n* = 6). The remaining 39 patients were processed for data analysis.

**Results:**

As pretreatment examinations, computed tomography (CT) was performed for the brain (*n* = 8), neck (*n* = 39), and chest (*n* = 32), magnetic resonance imaging (MRI) for the brain (*n* = 4) and neck (*n* = 23), positron emission tomography-CT (PET-CT) in 23 patients, bone scintigraphy in 4, neck ultrasonography in 9, and tumor markers in 25. Primary sites were oral cavity (*n* = 1), nasal cavity/paranasal sinuses (*n* = 16), nasopharynx (*n* = 2), oropharynx (*n* = 4), hypopharynx (*n* = 2), larynx (*n* = 6), salivary gland (*n* = 3), thyroid (*n* = 2), and others (*n* = 3). Stages were II/III/IV-A/IV-B/IV-C/Not determined = 3/5/16/6/5/4; stage IV comprised 69%. No patient had brain metastases. First-line treatments were divided into 3 groups: the chemoradiotherapy (CRT) group (*n* = 27), non-CRT group (*n* = 8), and best supportive care group (*n* = 4). The CRT group included concurrent CRT (CCRT) (*n* = 17), chemotherapy (Chemo) followed by radiotherapy (RT) (*n* = 5), and surgery (Surg) followed by CCRT (*n* = 5). The non-CRT group included Surg followed by RT (*n* = 2), Surg followed by Chemo (*n* = 1), RT alone (*n* = 2), and Chemo alone (*n* = 3). The 1-year/2-year overall survival (OS) of all 39 patients was 65.3/53.3%. The 1-year OS of the CRT group (77.6%) was significantly better compared with the non-CRT group (31.3%). There were no significant differences in adverse events between the CCRT group (*n* = 22) and the Chemo without concurrent RT group (*n* = 9).

**Conclusion:**

Neck and chest CT, neck MRI, and PET-CT would be necessary and sufficient examinations in the diagnostic set up for HNSmCC. CCRT may be recommended as the first-line treatment. The 1-year/2-year OS was 65.3%/53.3%. This study would provide basal data for a proposing the diagnostic and treatment algorithms for HNSmCC.

## Brief summary

Basal information of head and neck small-cell carcinoma (HNSmCC) remains sparse due to its rarity. We report a multicenter retrospective study of 39 patients with HNSmCC. Primary sites were oral cavity (*n* = 1), nasal cavity/paranasal sinuses (*n* = 16), nasopharynx (*n* = 2), oropharynx (*n* = 4), hypopharynx (*n* = 2), larynx (*n* = 6), salivary gland (*n* = 3), thyroid (*n* = 2), and others (*n* = 3). Stages were II/III/IV-A/IV-B/IV-C/Not determined = 3/5/16/6/5/4; stage IV comprised 69%. First-line treatments were divided into 3 groups: the chemoradiotherapy (CRT) (*n* = 27), non-CRT (*n* = 8), and best supportive care (*n* = 4). The CRT group included concurrent CRT (CCRT) (*n* = 17), chemotherapy (Chemo) followed by radiotherapy (RT) (*n* = 5), and surgery (Surg) followed by CCRT (*n* = 5). The non-CRT group included Surg followed by RT (*n* = 2), Surg followed by Chemo (*n* = 1), RT alone (*n* = 2), and Chemo alone (*n* = 3). The 1-year/2-year overall survival (OS) of all 39 patients was 65.3/53.3%. The 1-year OS of the CRT group (77.6%) was significantly better compared with the non-CRT group (31.3%). There were no significant differences in adverse events between the CCRT group (*n* = 22) and the Chemo without concurrent RT group (*n* = 9). It is suggested that CCRT may be recommended as the first-line treatment. This study would provide basal data for proposing the diagnostic and treatment algorithms for HNSmCC.

## Introduction

Small-cell carcinoma (SmCC) most commonly occurs in the lung (1), while head and neck (head/neck) small-cell carcinoma (HNSmCC) accounts for less than 1% of all SmCC (2, 3). Due to its rarity, comprehensive data regarding the epidemiology, primary site, optimal treatment, and prognosis remain unclear. In clinical practice, treatment for HNSmCC is often substituted by that for small-cell lung cancer (SCLC) due to histopathological similarities. However, therapeutic substitution by that for SCLC in the treatment of HNSmCC has not yet been verified (4–6). Large scale data are thus warranted, however, most reports on treatment outcomes in HNSmCC to date have included less than 20 patients (3, 7). In this multicenter study, therefore, we investigated the epidemiology, flow of diagnosis, treatment, and prognosis of HNSmCC using data of 39 patients from 10 participating institutions.

## Materials and methods

This study was conducted in accordance with the principles of the World Medical Association Declaration of Helsinki (1964) and the Ethical Guidelines for Medical and Health Research Involving Human Subjects (Ministry of Education, Culture, Sports, Science and Technology and Ministry of Health, Labor and Welfare Japan). The Niigata University Institutional Review Board approved the study (IRB2015-2685).

Patients with HNSmCC diagnosed at the following 10 institutions between January 2006 and December 2015 were enrolled in the study: Niigata University, Tokyo Medical University, Nara Medical University, National Cancer Center Central Hospital, Tokai University, Sapporo Medical University, Osaka University, Yokohama City University, Nippon Medical School, and International University of Health and Welfare Mita Hospital. No patient had any primary neoplastic disease in other organs including the lung. We retrospectively investigated information on age, sex, Eastern Cooperative Oncology Group Performance Status (ECOG PS), pretreatment examinations, pathological results, primary site, TNM classification, stage, treatment methods, treatment outcomes, adverse events, and prognosis. Pathological specimen was re-evaluated by central pathological judgment at the Department of Anatomic Pathology, Tokyo Medical University. Overall survival (OS) was compared among primary sites (nasal cavity/paranasal sinuses vs. others), different stages (Stage IV-C vs. others), and treatment regimens [chemoradiotherapy (CRT) vs. non-CRT]. OS was also compared among different CRT regimens (concurrent CRT (CCRT), chemotherapy (Chemo) followed by radiotherapy (RT), and surgery (Surg) followed by CCRT). The incidence of adverse events ≥Grade 3 was compared between the CCRT group (CCRT, Surg followed by CCRT) and the Chemo without concurrent RT group (Chemo followed by RT, Surg followed by Chemo, and Chemo alone).

Statistical analysis was performed using Statcel 3 statistical software (OMS Inc., Tokyo, Japan). For survival analyses, the Kaplan-Meier method was used to derive survival rate, and Fisher’s exact probability test was used to compare the incidence of adverse events.

## Results

Altogether, 47 patients with HNSmCC were enrolled in the study. Eight patients were excluded, those for whom no pathological specimens were available (*n* = 2) and those with discrepant central pathological judgements (*n* = 6, 2 patients with olfactory neuroblastoma and 1 each with adenoid cystic carcinoma, poorly differentiated carcinoma, undifferentiated carcinoma, and large-cell neuroendocrine carcinoma). Data for the remaining 39 patients were processed for analysis.

Table 1 shows demographics and clinical data of the 39 patients with HNSmCC. Age ranged from 34 to 89 (median 63) years with 31 males (79%) and 8 females (21%). ECOG PS was 0/1/2/3/unknown = 28/7/2/1/1, with 0 to 1 accounting for approximately 90%. Pathological diagnosis was made using biopsy specimens in 37 patients (95%), while HNSmCC was detected using postoperative pathology in 2 patients (5%). Primary sites were oral cavity (*n* = 1), nasal cavity/paranasal sinuses (*n* = 16), nasopharynx (*n* = 2), oropharynx (*n* = 4), hypopharynx (*n* = 2), larynx (*n* = 6), salivary gland (*n* = 3), thyroid (*n* = 2), and others (*n* = 3). The most common site was the nasal cavity/paranasal sinuses, accounting for 41%. Table 2 shows the Unio Internationalis Contra Cancrum (UICC) (8) TNM classification and stages. T2 (31%) and T4 (40%) in T-stage, and N0 (40%) and N2 (40%) in N-stage were the majority. M1 was found regardless of T and N-stage. Stage was II/III/IV-A/IV-B/IV-C/None = 3/5/16/6/5/4; Stage IV comprised 69%. Sites of distant metastases in Stage IV-C (*n* = 5) were axilla, liver, bone, esophagus with overlap, and multiple sites. Table 3 shows pretreatment examinations. Computed tomography (CT) was performed for the brain in 8 patients (21%), neck in 39 (100%), and chest in 32 (82%). Magnetic resonance imaging (MRI) was performed for the brain in 4 (10%) patients and neck in 23 (59%). Positron emission tomography-CT (PET-CT) was performed in 23 patients (59%), bone scintigraphy in 4 (10%), and neck ultrasonography in 9 (23%). For tumor markers, neuron-specific enolase (NSE) and pro-gastrin-releasing peptide (ProGRP) (9) were measured in 25 patients and 22, respectively. Of these, 13/25 (52%) and 4/22 (18%) were above normal limits, respectively.

**Table 1:** Baseline patient demographics and clinical data.

	*n*	(%)
**Age (years)**	34–89	(Median 63)
**Sex**	
Male	31	(79%)
Female	8	(21%)
**ECOG PS**	
0	28	(71%)
1	7	(18%)
2	2	(5%)
3	1	(3%)
Unknown	1	(3%)
**Pathological diagnosis method**	
Biopsy	37	(95%)
Aspiration	0	(0%)
Postoperative pathology	2	(5%)
**Primary site**	
Oral cavity	1	(3%)
Nasal cavity/paranasal sinuses	16	(41%)
Nasopharynx	2	(5%)
Oropharynx	4	(10%)
Hypopharynx	2	(5%)
Larynx	6	(15%)
Salivary gland	3	(8%)
Thyroid	2	(5%)
Others	3	(8%)

ECOG PS, Eastern cooperative oncology group performance status.

**Table 2:** TNM classification and stage of patients.

TNM classification					
	N0	N1	N2	N3	
T1			2		2 … 6%
T2	2	3 (1)	5	1 (1)	11 (2) … 31%
T3	4 (1)		4 (1)		8 (2) … 23%
T4	8	3	3 (1)		14 (1) … 40%
	14 (1) … 40%	6 (1) … 17%	14 (2) … 40%	1 (1) … 3%	35 (5)
Number of parentheses indicates M1 patient					
		*n*	(%)		
Stage					
II		3	(8%)		
III		5	(13%)		
IV-A		16	(41%)	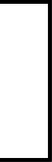	Stage IV: 27 (69%)
IV-B		6	(15%)		
IV-C		5	(13%)		
None		4	(10%)		

**Table 3:** Pretreatment examinations.

	*n*	(%)
**CT**		
Brain	8	(21%)
Neck	39	(100%)
Chest	32	(82%)
**MRI**		
Brain	4	(10%)
Neck	23	(59%)
PET-CT	23	(59%)
Bone Scintigraphy	4	(10%)
Ultrasonography of neck	9	(23%)
Tumor marker	Above normal/Number examined	
NSE	13/25	(52%)
ProGRP	4/22	(18%)
SCC	7/22	(32%)
CEA	6/19	(32%)
CYFRA	2/11	(18%)
CA19-9	0/6	(0%)
SLX	1/5	(20%)

CT, computed tomography; MRI, magnetic resonance imaging; PET-CT, positron emission tomography-CT; NSE, neuron-specific enolase; ProGRP, pro-gastrin-releasing peptide; SCC, squamous cell carcinoma; CEA, carcinoembryonic antigen; SLX, Sialyl Lewis X.

Table 4 shows the first-line treatment and outcomes. First-line treatment were CCRT (*n* = 17), Chemo followed by RT (*n* = 5), Surg followed by CCRT (*n* = 5), Surg followed by RT (*n* = 2), Surg followed by Chemo (*n* = 1), RT alone (*n* = 2), Chemo alone (*n* = 3), and best supportive care (BSC) (*n* = 4). CCRT was the most common accounting for 43%. Surg was performed in 8 patients (21%), all of whom received postoperative treatment (CCRT 5, RT 2, Chemo 1). The CRT group included 27 patients who received CCRT, Chemo followed by RT, or Surg followed by CCRT accounting for 69%. Eight patients in the non-CRT group received Surg followed by RT, Surg followed by Chemo, RT alone, or Chemo alone, accounting for 21%. Response evaluation according to the Response Evaluation Criteria in Solid Tumors version 1.1 was Complete response (CR)/Partial response (PR)/Progressive disease (PD)/Indeterminate = 21/6/7/5. Eighteen of the 21 CR and 5 of the 6 PR patients had received CRT.

**Table 4:** First-line treatment and outcomes.

	*n*	(%)		
**Treatment**				
CCRT	17	(43%)	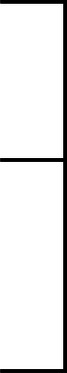	CRT 27 (69%)
Chemo followed by RT	5	(13%)		
Surg followed by CCRT	5	(13%)		
Surg followed by RT	2	(5%)		Non-CRT 8 (21%)
Surg followed by Chemo	1	(3%)		
RT alone	2	(5%)		
Chemo alone	3	(8%)		
BSC	4	(10%)		
**Outcome: response evaluation according to the RECIST version 1.1**				
CR	21	(54%)	…	CRT 18/21
PR	6	(15%)	…	CRT 5/6
PD	7	(18%)		
Indeterminate	5	(13%)		

CCRT, concurrent chemoradiotherapy; Chemo, chemotherapy; RT, radiotherapy; Surg, surgery; CRT, chemoradiotherapy; BSC, best supportive care; RECIST, response evaluation criteria in solid tumors; CR, complete response; PR, partial response; PD, progressive disease.

Table 5 shows irradiation methods and chemotherapy regimens. In RT, radical irradiation was performed in 31 patients at a dose of 50 to 70 Gy, with a median of 60 Gy. Irradiation was delivered using standard fractionation (SF) 2 Gy/fraction (Fr) in 25 patients, SF 1.8 Gy/Fr in 4, and accelerated hyperfractionation (AHF) 1.5 Gy/Fr in 1. Chemo was administered in 31 patients with overlap, 22 of which were for CCRT. The CCRT Chemo regimen was Cisplatin (CDDP) or Carboplatin (CBDCA) + Etoposide (ETP) in 18 of 22 patients, CDDP or CBDCA + Irinotecan (CPT-11) in 3, and CDDP + Fluorouracil (5-FU) in 1. Overall, 1 to 6 courses of Chemo were administered in CCRT; 4 courses were the most frequent with a median of 3 courses. For Chemo alone, CDDP + CPT-11 or ETP was used for 1 and 3 patients, respectively. The only use of immune checkpoint inhibitor (ICI) was nivolumab for 1 out of 39 patients.

**Table 5:** Irradiation method and chemotherapy regimen.

	*n*
**Irradiation method**	
Number of patients who underwent radical irradiation … 31	
CCRT	17
Chemo followed by RT	5
Surg followed by CCRT	5
Surg followed by RT	2
RT alone	2
Irradiation dose … 50–70 Gy (Median 60 Gy)	
50 (Gy)	4
50.4	2
54	2
59.4	1
60	13
66	3
70	4
Halfway completed (due to disease progression)	1
Unknown	1
Irradiation delivery mode	
Standard fractionation 2Gy/Fr	25
Standard fractionation 1.8Gy/Fr	4
Accelerated hyperfractionation 1.5Gy/Fr	1
Unknown	1
**Chemotherapy regimen**	
Number of patients who underwent Chemo … 31 (with overlap)	
CDDP + ETP	20
CBDCA + ETP	8
CDDP + CPT-11	3
CBDCA + CPT-11	1
CPT-11	1
CDDP + 5-FU	1
PTX + C-mab	1
Nivolumab	1
Number of patients who underwent CCRT … 22	
CDDP + ETP	13
CBDCA + ETP	5
CDDP + CPT-11	2
CBDCA + CPT-11	1
CDDP + 5-FU	1
Number of courses of Chemo in CCRT … 1–6 (median 3)	
6	1
4	8
3	5
2	4
1	4

CCRT, concurrent chemoradiotherapy; Chemo, chemotherapy; RT, radiotherapy; Surg, surgery; Fr, fraction; CDDP, Cisplatin; ETP, Etoposide; CBDCA, Carboplatin; CPT-11, Irinotecan; 5-FU, Fluorouracil; PTX, Paclitaxel; C-mab, Cetuximab.

**Figure 1:**
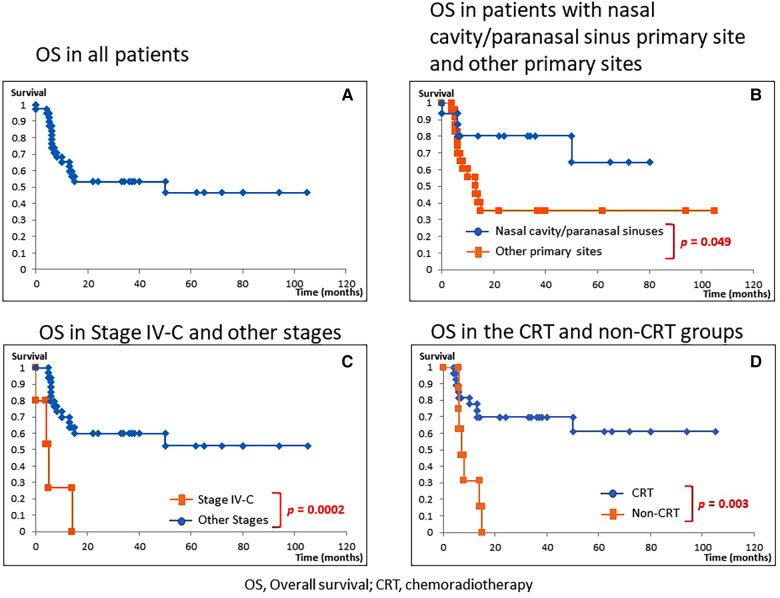
Overall survival (OS). (**A**) OS in all patients. (**B**) OS in patients with nasal cavity/paranasal sinuses diseases vs. other primary sites. (**C**) OS in Stage IV-C vs. other stages. (**D**) OS in the chemoradiotherapy (CRT) group vs. non-CRT group. The 1-year/2-year OS of all patients was 65.3/53.3% (median 14 months). (**A**) For patients with nasal cavity/paranasal sinuses diseases, the 1-year/2-year OS was 80.4/80.4% (median 29 months), which demonstrated a significantly better prognosis than those of patients with primary sites other than the nasal cavity/paranasal sinuses (*P* = 0.049, **B**). For Stage IV-C patients, the 1-year OS was 26.7% (median 4 months), demonstrating a significantly poorer prognosis than for those with other than Stage IV-C (*P* = 0.0002, **C**). For the CRT group, 1-year/2-year OS was 77.6/69.8% (median 22 months), demonstrating a significantly better prognosis than for those in the non-CRT group (*P* = 0.003, **D**).

Figure 1 shows OS rates. The observation period was 0 to 105 months, with a median of 14 months. Because there was only 1 mortality from other causes, survival rate was evaluated using only OS. The 1-year/2-year OS of all patients was 65.3/53.3%; median survival was 14 months (Figure 1A). For patients whose primary site was the nasal cavity/paranasal sinuses, the most common site in this study, 1-year/2-year OS was 80.4/80.4% with a median of 29 months. This was significantly better than that for those patients with other primary sites than the nasal cavity/paranasal sinuses (*P* = 0.049, Figure 1B). For Stage IV-C patients, 1-year OS was 26.7% with a median survival of 4 months demonstrating significantly worse prognosis than that for patients with other stages than Stage IV-C (*P* = 0.0002, Figure 1C). For the CRT group, 1-year/2-year OS was 77.6/69.8% with a median survival of 22 months demonstrating significantly better prognosis than those in the non-CRT group (*P* = 0.003, Figure 1D).

**Figure 2:**
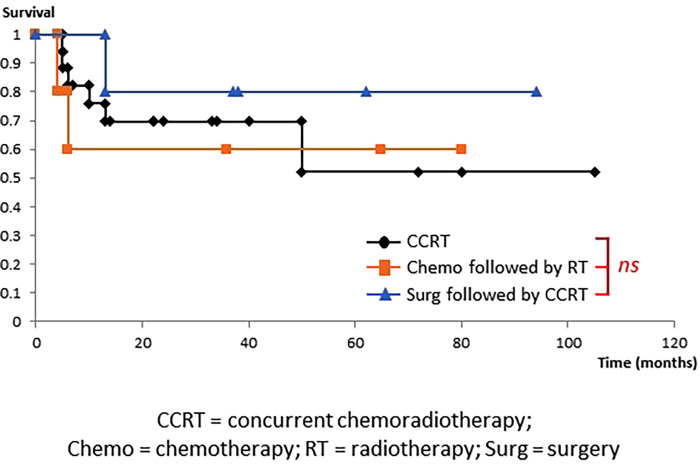
Overall survival in the chemoradiotherapy group. OS did not differ among the three treatment groups, namely, concurrent chemoradiotherapy (CCRT), chemotherapy (Chemo) followed by radiotherapy (RT), and surgery (Surg) followed by CCRT.

Figure 2 shows OS of each treatment in the CRT group. There were no significant differences in OS among the three groups (CCRT, Chemo followed by RT, and Surg followed by CCRT).

Table 6 shows the incidence of grade ≥3 adverse events according to the Common Terminology Criteria for Adverse Events version 4.0 for 22 patients with the CCRT group (CCRT (*n* = 17), Surg followed by CCRT (*n* = 5)) and 9 patients with the Chemo without concurrent RT group (Chemo followed by RT (*n* = 5), Surg followed by Chemo (*n* = 1), Chemo alone (*n* = 3)). Although mucositis tended to be more frequent in the CCRT group (*P* = 0.08), there was no significant difference in all adverse events between the CCRT group and Chemo without concurrent RT group.

**Table 6:** Adverse events according to the CTCAE version 4.0 in the concurrent chemoradiotherapy and chemotherapy without concurrent radiotherapy groups.

	CCRT (*n* = 22)			Chemo without concurrent RT (*n* = 9)			*P*-value for incidence of adverse event (Fisher’s exact test)
	grade 3	grade 4	Total	grade 3	grade 4	Total	
Myelosuppresion	8	10	18 (81.8%)	1	5	6 (66.7%)	0.36
Febrile neutropenia	3	1	4 (18.2%)	1	0	1 (11.1%)	0.63
Acute renal dysfunction	(grade 1: 1)		0 (0.0%)	(grade 1: 1)		0 (0.0%)	—
Hyponatremia	0	1	1 (4.5%)	0	1	1 (11.1%)	0.50
Nausea vomiting	2	1	3 (13.6%)	0	0	0 (0.0%)	0.24
Liver dysfunction	1	0	1 (4.5%)	0	0	0 (0.0%)	0.52
Mucositis	6	0	6 (27.3%)	0	0	0 (0.0%)	0.08
Diarrhea	1	0	1 (4.5%)	0	0	0 (0.0%)	0.52
Delirium	0	0	0 (0.0%)	0	1	1 (11.1%)	0.11

CTCAE, common terminology criteria for adverse events; CCRT, concurrent chemoradiotherapy; Chemo, chemotherapy; RT, radiotherapy.

In summary, CCRT was the most common treatment with better outcomes compared to other treatment regimens and with comparable adverse events.

## Discussion

### SCLC and HNSmCC

**Figure 3:**
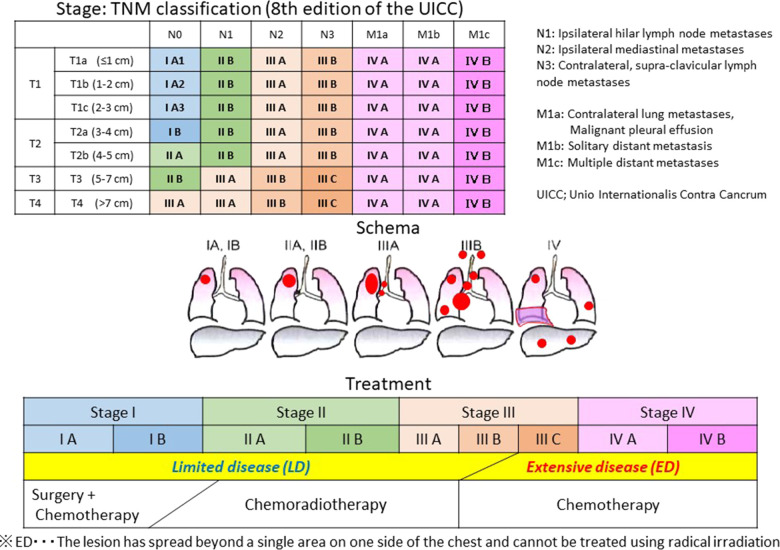
Stage and treatment of small-cell lung carcinoma. CRT is administered for the limited disease (LD), where the lesion is contained in a single area on one side of the chest for which radical irradiation is possible. Chemotherapy is used for the extensive disease (ED), where the lesion has spread beyond a single area and cannot be treated using radical irradiation.

**Figure 4:**
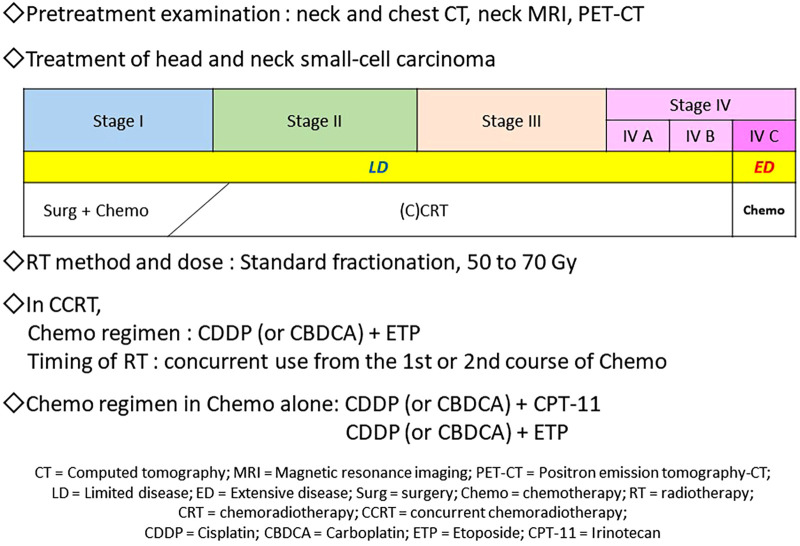
Proposal of the diagnostic and treatment algorithm of head and neck small-cell carcinoma. Regarding pretreatment examinations, neck and chest CT, neck MRI, and PET-CT are necessary and sufficient for deciding stages and ruling out small-cell lung carcinoma. For the first-line treatment, CRT, especially CCRT is recommended for Stage ≤IV-B disease; RT 60–70 Gy by standard fractionation and Chemo with CDDP or CBDCA + ETP (4 courses) may be administered. Regarding the timing of RT, concurrent use from the first or second course of Chemo may be recommended. In Stage IV-C, Chemo alone, such as the CDDP or CBDCA + CPT-11 or ETP regimens, is recommended.

SmCC is a neuroendocrine tumor pathologically representing a proliferative type with neuroendocrine differentiation (10, 11) of which 96% are SCLC that occurs in the lung (1). SCLC is highly sensitive to RT and Chemo, therefore, CRT is often administered in the limited disease (LD), where the lesion is contained in a single area on one side of the chest for which radical irradiation is possible. Chemo is applied to the extensive disease (ED), where the lesion has spread beyond a single area and cannot be treated using radical irradiation (Figure 3) (12, 13). While there are no well-established standardized diagnostic and treatment algorithms for HNSmCC as for SCLC, treatment for HNSmCC is often substituted by that for SCLC (1, 3–5, 14). For the selection of treatment method, treatment strategy for each SCLC stage is converted to that for HNSmCC (Figure 4) (1, 3–5, 15–17). According to this, patients indicated for Surg are quite limited. CRT is recommended for up to Stage ≤IV-B for which RT is indicated, while Chemo alone is recommended for Stage IV-C.

### Patients and examinations

Among the 47 patients initially enrolled in this study, 6 (13%) were diagnosed as not having HNSmCC. The 4th edition of the World Health Organization Classification of Tumors of the Lung, Pleura, Thymus and Heart recommends performing immunohistochemistry to confirm neuroendocrine differentiation (per chromogranin A, synaptophysin, and CD 56 staining), epithelial differentiation (per cytokeratin staining such as AE 1/AE 3, CAM 5.2, and MNF 116), and high proliferative activity (per Ki-67 index) (11, 18–20). Central pathological judgements in this study performed these immunohistochemistry if indicated, suggesting an importance of accurate pathological diagnosis of HNSmCC.

Regarding imaging examinations, it is essential to evaluate the local spread of the tumor and detect a possible primary lesion in the lung (8, 15), and whole-body PET-CT scan is likely being more commonly used than bone scintigraphy (5, 16). While no patients had brain metastases in this study, the usefulness of tumor markers for the aid in diagnosis, monitoring of treatment effects and recurrences could not be confirmed (Table 3) (9). Consequently, neck and chest CT, neck MRI, and PET-CT would be necessary and sufficient examinations for deciding stages and ruling out SCLC.

As for the primary sites, nasal cavity/paranasal sinuses (*n* = 16, 41%) was remarkably more often than previous reports (Table 1) (4, 14, 21, 22). As for stage, stage IV (*n* = 27, 69%) was as many as other reports (3, 11, 12, 21, 22), and Stage IV-C (*n* = 5, 13%) was less than other reports (Table 2) (3, 21, 22).

### Treatment outcomes

The 1-year/2-year OS of all patients was 65.3/53.3% (Figure 1), which was considerably better than that of previous reports (1, 3–5, 14). These favorable results could be attributed to the high proportion of nasal cavity/paranasal sinus disease (Table 1, Figure 1B), which is known to have a good prognosis (23), and also to the small number of Stage IV-C disease (Table 2, Figure 1C) (1, 3, 7, 21, 22).

As for the first-line treatment, Surg was performed in 8 patients, all of them received postoperative treatment. Therefore, first-line treatment methods were divided into 3 groups: CRT, non-CRT, and BSC (Table 4). CRT was often selected as the first-line treatment for HNSmCC substituted by that for SCLC in previous reports (1, 3–5, 14, 16, 22). In this study, CRT was also selected for 69% of patients with good outcome (Figure 1D).

There were no significant differences in treatment outcomes among different CRT regimens (Figure 2). Moreover, incidence of adverse events did not differ between the CCRT group (*n* = 22) and Chemo without concurrent RT group (*n* = 9) (Table 6), suggesting that CCRT, which can be completed within a shorter treatment period than other CRT regimens, may be recommended as the first-line treatment for HNSmCC (Figure 4).

Regarding the irradiation method in the CCRT group, 50 to 70 Gy with a median of 60 Gy by SF was used, which is also common for the head/neck region. However, 45 Gy by AHF is recommended for the treatment of SCLC (24). These doses and methods are determined based on the tolerable dose for each organ, hence the differences in dose and irradiation method between head/neck and lung are plausible. For the Chemo regimen in CCRT, 4 courses of CDDP or CBDCA + ETP, as recommended in SCLC (13, 24), were used in 18 out of 22 patients. Consistent with other reports, regimens used in this study were apparently selected based on those for SCLC (1, 3–5, 14, 16, 22).

Data on the timing of concurrent use of Chemo and RT in CCRT, that is, from the first or second course, could not be obtained. In SCLC, it is recommended to start RT concurrently with the first course of Chemo (25), while concurrent RT from the second course was also used (26). If therapeutic planning for RT to head/neck is prolonged, it may be better not to schedule starting concurrently from the first course and to preferably introduce 1 course of Chemo first and introduce CCRT from the second course.

For the Chemo alone regimen, CDDP + CPT-11 or ETP was used for 1 and 3 patients, respectively. This also seemed to be selected based on SCLC (17, 27). For ICI therapy, atezolizumab and durvalumab have been approved for SCLC (28, 29). Nonetheless, the only use of ICI in this study was nivolumab for 1 patient; ICI use for HNSmCC might increase in the future.

### Proposal of the diagnostic and treatment algorithm

Below is a proposal of the diagnostic and treatment algorithm derived from this study (Figure 4). Regarding the pretreatment examinations in HNSmCC, neck and chest CT, neck MRI, and PET-CT are necessary and sufficient for deciding stages and ruling out SCLC. For the first-line treatment, CRT, especially CCRT is recommended for Stage ≤IV-B disease; RT 50 to 70 Gy by SF and Chemo with CDDP or CBDCA + ETP (4 courses) may be administered. Regarding the timing of RT, concurrent use from the first or second course of Chemo may be recommended. For Stage IV-C, Chemo alone, such as the CDDP or CBDCA + CPT-11 or ETP regimens, is recommended.

### Limitation of the study

This study involved considerably larger sample sizes compared to previous reports, nevertheless, meta-analyses or systematic reviews are requisite to establish optimal diagnostic and treatment algorithms for HNSmCC.

## Conclusions

We examined 39 patients with HNSmCC during a 10-year period from 10 participating institutions. The most common primary site was the nasal cavity/paranasal sinuses (*n* = 16), accounting for 41%. Neck and chest CT, neck MRI, and PET-CT were useful for deciding stages and ruling out SCLC. CRT was performed as the first-line treatment in 27 patients (69%) which included 17 CCRT (43%). Treatment outcomes were significantly better in the CRT group than the non-CRT group. The incidence of adverse events did not differ between the CCRT group and the Chemo without concurrent RT group, therefore, CCRT may be recommended as the first-line treatment. The 1-year/2-year OS was 65.3%/53.3%. This study would provide basal data for proposing the diagnostic and treatment algorithms for HNSmCC.

## Data Availability

The original contributions presented in the study are included in the article/Supplementary Material, further inquiries can be directed to the corresponding author/s.
